# Detecting high-risk neighborhoods and socioeconomic determinants for common oral diseases in Germany

**DOI:** 10.1186/s12903-024-03897-4

**Published:** 2024-02-09

**Authors:** Sebastian Völker, Antje van der Zee-Neuen, Alexander Rinnert, Jessica Hanneken, Tim Johansson

**Affiliations:** 1 Data Science Center of Excellence, BFS health finance, Bertelsmann, Dortmund, Germany; 2 Center for Public Health and Healthcare Research, Institute of General Practice, Family Medicine and Preventive Medicine, Program Medical Science, Paracelsus Medical University, Salzburg, Austria; 3 Center for Physiology, Pathophysiology and Biophysics, Institute for Physiology and Pathophysiology/Gastein Research Institute/Center for Public Health and Healthcare Research, Paracelsus Medical University, Salzburg, Austria; 4 Ludwig Boltzmann Institute for Arthritis and Rehabilitation, Salzburg, Austria; 5 Healthcare & Politics, BFS health finance, Bertelsmann, Dortmund, Germany; 6 Salzburg Regional Health Fund, SAGES, Salzburg, Austria

**Keywords:** Spatial cluster analysis, Incidence rate, Socioeconomic determinants of health, Dental health, Artificial intelligence, Machine learning, Geostatistics

## Abstract

**Background:**

Ideally, health services and interventions to improve dental health should be tailored to local target populations. But this is not the standard. Little is known about risk clusters in dental health care and their evaluation based on small-scale, spatial data, particularly among under-represented groups in health surveys. Our study aims to investigate the incidence rates of major oral diseases among privately insured and self-paying individuals in Germany, explore the spatial clustering of these diseases, and evaluate the influence of social determinants on oral disease risk clusters using advanced data analysis techniques, i.e. machine learning.

**Methods:**

A retrospective cohort study was performed to calculate the age- and sex-standardized incidence rate of oral diseases in a study population of privately insured and self-pay patients in Germany who received dental treatment between 2016 and 2021. This was based on anonymized claims data from BFS health finance, Bertelsmann, Dortmund, Germany. The disease history of individuals was recorded and aggregated at the ZIP code 5 level (*n* = 8871).

**Results:**

Statistically significant, spatially compact clusters and relative risks (RR) of incidence rates were identified. By linking disease and socioeconomic databases on the ZIP-5 level, local risk models for each disease were estimated based on spatial-neighborhood variables using different machine learning models. We found that dental diseases were spatially clustered among privately insured and self-payer patients in Germany. Incidence rates within clusters were significantly elevated compared to incidence rates outside clusters. The relative risks (RR) for a new dental disease in primary risk clusters were min = 1.3 (irreversible pulpitis; 95%-CI = 1.3–1.3) and max = 2.7 (periodontitis; 95%-CI = 2.6–2.8), depending on the disease. Despite some similarity in the importance of variables from machine learning models across different clusters, each cluster is unique and must be treated as such when addressing oral public health threats.

**Conclusions:**

Our study analyzed the incidence of major oral diseases in Germany and employed spatial methods to identify and characterize high-risk clusters for targeted interventions. We found that private claims data, combined with a network-based, data-driven approach, can effectively pinpoint areas and factors relevant to oral healthcare, including socioeconomic determinants like income and occupational status. The methodology presented here enables the identification of disease clusters of greatest demand, which would allow implementing more targeted approaches and improve access to quality care where they can have the most impact.

**Supplementary Information:**

The online version contains supplementary material available at 10.1186/s12903-024-03897-4.

## Background

One in two adults in the EU (European Union) suffers from at least one oral disease [1]. Oral diseases, most of which are chronic and progressive in nature, can cause pain, infection and low quality of life, because they impede essential functions, such as eating and speaking. However, they may also affect psychosocial and general health, and prevent individuals from participating in society [1].

Periodontitis, caries, pulp diseases, and tooth loss are the most important oral diseases in Europe and worldwide. Many oral diseases are largely preventable at an early stage [2]. Although recent improvements in oral health have been observed [3], oral diseases remain highly prevalent. A significant part of the disease burden is associated with socioeconomic status, age, and lifestyle behavior, as is the case for many chronic conditions (diabetes, obesity, heart disease, etc.) [2].

In 2021, the World Health Assembly approved a historic WHO resolution on oral health, mandating all member states to address key risk factors of oral diseases, enhance the capacity of oral health professionals to deliver consistent and high quality care, foster the shift from a traditional curative approach towards a preventive approach, and better integrate strategies on oral health promotion, prevention and treatment [4].

Surveys to measure the prevalence and incidence of oral diseases and their impact on quality of life are key to many public health systems. They aid policy makers in making decisions on priority areas and the allocation of resources to address those priorities. However, it is well established that certain community groups in some areas suffer a higher burden of disease [2]. These groups are usually under-represented in surveys and a closer examination is necessary in order to facilitate the work of policy makers and service planners in targeted approaches [5]. While dental care in Germany is good, there is still insufficient attention paid to how and where dental diseases arise.

In general, the neighborhood in which people live profoundly influences social and health behaviors [6]. Spatial epidemiology focuses explicitly on analyzing spatial aspects of health-related data, taking into account demographic, environmental, socioeconomic, genetic, and behavioral risk factors to describe spatial distributions, discover patterns of spatial association (also known as spatial clustering), and detect spatial instability and atypical observations. The detection of spatial associations (clustering) enables the derivation of local or neighborhood spatial associations (clusters) [6]. The basic approach to spatial epidemiology goes back to Tobler’s First Law of Geography, in which he describes that “everything is related to everything else, but near things are more related than distant things” [7].

Although spatial cluster analysis in health care research has been used in some studies [8–10] and Nayak et al. [11] have described its potential applications, the use of these methods have remained uncommon in oral health care research. Most studies have been conducted in Brazil, where spatial patterns of tooth decay and dental treatment needs in 5–12-year-old schoolchildren [12], DMFT (decayed missing filled teeth) index of 12-year-old schoolchildren [13], gender differentials in the distribution of dental caries and restorative treatment in 11- and 12-year-old girls and boys [14], and oral cancer [15] were examined. These studies revealed that dental health services are less used in neighborhoods with a poorer socioeconomic status. Importantly, specific neighborhoods could be identified for oral public health interventions. A study from Braunschweig, Germany, identified spatial disparities in the DMFT index of children aged 3–6 years visiting a daycare center between 2009 and 2014. Significant spatial clustering of DMFT was assessed and spatial clusters were identified on a city district level. In a spatial lag model, sociodemographic characteristics, particularly the proportion of unemployed persons and proportion of persons with a migration background, were significantly associated with a higher DMFT index [16].

While these studies provide relevant insight into specific age groups and gender-related differences in oral diseases, evidence on small-scale spatial disparities and of the social determinants of health that describe spatial- and population-specific needs in dental care remains rare. Additionally, many studies in health care focus on publicly insured patients. However, in Germany, 12% of the entire population are not publicly insured. Moreover, in dental care, there is also a considerable number of publicly insured self-payers (the revenue of dental practices from privately insured individuals and self-payers in Germany amounts to 50%), who are also excluded from, e.g. secondary data analysis. Nowadays, new options for data analysis are available, but the possibilities of applying such technologies to the analysis of claims data in oral health research and practice still needs to be established and promoted.

Here, we aimed to explore the incidence rate of the oral diseases periodontitis, severe caries, irreversible pulpitis, and tooth loss in treated patients (privately insured and self-payers) in Germany from 2016 to 2021 using data from a leading private provider of financial services in the healthcare sector. We further investigated whether oral diseases are spatially clustered in Germany, and whether their clustering can be explained by neighborhood socioeconomic determinants.

The following null hypotheses were addressed:

1. the incidence rates of privately insured and self-payers with the oral diseases periodontitis, severe caries, irreversible pulpitis, and tooth loss in Germany in the period between 2016 and 2021 are not comparable to other studies on incidence rates of these diseases;
2. spatial dimension is not an important determinant of the oral diseases, and oral diseases are not spatially clustered in Germany;
3. primary oral disease risk clusters cannot be explained by social determinants of health using machine learning models.

Accordingly, the objectives of our study were delineated as follows:

1. Investigation of incidence rates among privately insured and self-paying individuals: This objective entails assessing and comparing the incidence rates of key oral diseases - namely periodontitis, severe caries, irreversible pulpitis, and tooth loss - among privately insured and self-paying populations in Germany over the period from 2016 to 2021.
2. Examination of the spatial distribution of oral diseases across Germany: This aspect of the study focuses on analyzing the spatial dimension as a potential determinant in the distribution of oral diseases. It aims to ascertain whether conditions like periodontitis, severe caries, irreversible pulpitis, and tooth loss demonstrate any spatial clustering within Germany.
3. Evaluation of the role of social determinants in oral disease risk clusters: The final objective is centered on employing machine learning models to scrutinize the extent to which social determinants of health can explain the formation of primary oral disease risk clusters in the German population.

## Methods

### Data sources and study population

This study is a retrospective cohort analysis utilizing anonymized claims data from privately insured and self-pay individuals who underwent dental treatment in Germany over a six-year period, from January 1, 2016, to December 31, 2021. The data was sourced from BFS health finance, a division of Bertelsmann, located in Dortmund, Germany, which is a leading provider of financial services in the healthcare sector.

For each participant in the study, we meticulously extracted and analyzed a range of data points. These included detailed information on disease-specific dental treatments received, the dates of these treatments, the age of the patient at the time of their last disease-specific treatment, and their five-digit postal code (ZIP-5) at the time of both the first and last treatments. This ZIP-5 coding system is crucial in providing a granular geographical context for our analysis, enhancing the spatial dimension of our study.

To ensure precise identification and categorization of treatments, we relied on the figures from the German dental fee schedule (Gebührenordnung für Zahnärzte, GOZ). This fee schedule is a comprehensive system used in Germany to standardize dental treatment charges and is crucial for identifying specific types of dental treatments in a consistent and systematic manner. To further bolster the reliability and accuracy of our disease estimation, we established specific inclusion and exclusion criteria based on this fee schedule. These criteria, detailed in the Appendix of our report, serve as a robust framework for accurately assessing the prevalence and characteristics of oral diseases among the study population.

There was a distortion in the distribution of individuals according to ZIP-5, which necessitated the use of an empirical Bayesian smoothing technique [17]. Local Empirical Bayesian Smoothing uses the population in a region as a measure of the confidence in the data, with higher populations in a given area lending a higher confidence to the estimated number of events in that location. Based on a rook neighborhood network, estimates for areas with low margins of error were left out, but estimates in regions with high margins of error were nudged closer to the global average of the event rate.

#### Computation of incidence rates

The incidence rate is a measure of the frequency at which new cases of a condition occur in a population over a specified period of time. In this manuscript the terms incidence rate and treatment incidence rate are used interchangeably. To calculate the incidence rates, the global and local incidence rates for each oral disease was assessed based on the study population. This was achieved by dividing the number of new cases of oral disease treatment during the entire study period by the time each person was observed (person-time), summed for all persons. Person-time was calculated for each individual, accounting for persons lost to follow-up or who died during the study period. Individuals were aggregated at the ZIP-5 level (*n* = 8871). For each year and the entire study period, incidence rates were calculated globally and locally at the ZIP-5 for each oral disease.

The exact Poisson 95%-Confidence Interval for the true diseases incidence rates was used [18]. Based on the link between the chi-square distribution and the Poisson distribution, exact Poisson confidence limits were calculated as the Poisson means divided by person-time, for distributions with the observed number of events and probabilities relevant to the confidence level (Eq. 1 and 2).

1 $${Y}_l=\frac{\upchi_{2Y,\alpha /2}^2}{2}$$

2 $${Y}_u=\frac{\upchi_{2\left(Y+1\right),1-\alpha /2}^2}{2}$$

where *Y* is the observed number of events, *Y*_l_ and *Y*_*u*_ are lower and upper confidence limits for *Y*, respectively, *χ*^*2*^_*v,a*_ is the chi-square quantile for upper tail probability on *v* degrees of freedom.

To calculate standardized rates, direct age and sex standardization was applied [19] to the 2013 European Standard Population [20] using 5-year age bands up to 90 years of age. Population data from the German Census 2011 1km^2^ grid-cells [21], were used to create population-weighted centroids for each ZIP-5. The raster cells were grouped by ZIP code areas and the arithmetic centroid was calculated from the average X and Y values of their geometric centroid and weighted by population in the raster cells according to Eq. 3.

3 $${\overline{X}}_{\omega }=\frac{\sum_{i=1}^n{\omega}_i{x}_i}{\sum_{i=1}^n{\omega}_i},{\overline{Y}}_{\omega }=\frac{\sum_{i=1}^n{\omega}_i{y}_i}{\sum_{i=1}^n{\omega}_i}$$

where ${\overline{X}}_{\omega }$ and ${\overline{Y}}_{\omega }$ represents the weighted X and Y coordinates of the ZIP code district, n represents the number of grid cells within the ZIP code district, *x*_*i*_ and *y*_*i*_ represents the X and Y coordinates of the geometric centroid of the grid cell in question, and *ω*_*i*_ represents the population size of the grid cell.

#### Detection of spatial risk clusters

To detect spatial risk clusters, we first calculated global Moran’s *I* to assess whether the incidence rates (per 1000 population) for each oral disease in German ZIP-5 neighborhoods displayed a tendency to cluster together, and to measure the extent of the correlation among neighboring observations. Global Moran’s index is used to examine the absence or presence of spatial autocorrelation in disease diffusion processes by comparing location and attribute similarities in the area [22, 23]. The global Moran’s *I* value must demonstrate a clustering distribution pattern in order to determine high or low-risk clusters for further analysis [24]. The formula for calculating global Moran’s *I* index is shown in Eq. 4:

4 $$I=\frac{\frac{\sum_i{\sum}_j{w}_{ij}{Z}_i{Z}_j}{S_0}}{\frac{\sum_i{Z}_i^2}{n}}$$

where *Zi* and *Zj* represents the incidence rate of each oral disease variations in neighborhood *i* and *j*, respectively, *w*_*ij*_ refers to the elements in the spatial weights matrix neighborhood *i* and *j* at study period, *S*0 = Σ_*i*_ Σ_*j*_*w*_*ij*_, *w*_*ij*_ as the sum of all weights, and n represents the number of observations.

The calculated global Moran’s *I* can range between − 1.0 and + 1.0, where a positive value suggests the presence of a positive spatial correlation, while a negative value suggests a negative correlation. Positive spatial autocorrelation means that geographically nearby values of a variable tend to be similar on a map, i.e. high values tend to be located near high values, medium values near medium values, and low values near low values [25]. Values close to 0 indicate a lack of spatial autocorrelation and that the distribution of data is random [26].

The local Moran’s *I*, a local indicator of spatial association (LISA), was then used to evaluate the local level of spatial autocorrelation or dependency of spatial data, and to visualize possible high-risk or low-risk clusters [27] based on incidence rates of each oral disease in the different neighborhoods.

The formula [27] for calculating local Moran’s index *I*_*i*_ is shown in Eq. 5:

5 $${I}_i={x}_i{\sum}_j{w}_{ij}{x}_j$$

where *x*_*i*_ and *x*_*j*_ represents the incidence rates of each oral disease in neighborhood *i* and *j* respectively, *w*_*ij*_ is the spatial weights matrix.

Global and local Moran’s *I* tests were performed using the first-order queen’s contiguity spatial weights matrix, which considers the values from all first-order neighborhoods to assess the degree of spatial autocorrelation by determining whether an area has a higher or lower mean compared to neighboring areas. The local Moran’s *I* divided the neighborhood polygons into four categories: high-high (hotspots), low-low (coldspots), high-low, and low-high, based on the type of spatial autocorrelations [27]. The high-high and low-low areas represent spatial clusters, whereas the high-low and low-high areas represent discordant patterns. For each point, the intensity value was calculated to indicate the degree of clustering of similar values around the given point. The local Moran’s *I* results, showing local spatial autocorrelation of oral disease incidences in the German ZIP-5 neighborhoods, were presented as cluster maps and Moran scatterplots [28]. Additionally, Bonferroni correction was performed to assess the robustness of the findings. However, with application of the Bonferroni bounds, no low-low and high-high clusters were found to be significant. As our goal was to test if further local cluster investigations were applicable rather than implementing the most stringent tests, we chose to employ the traditional *p*-value cut-off of 0.05.

Kulldorff’s scan statistic method [29] was used to identify spatial clusters for each oral disease between January 2016 and December 2021 in German ZIP-5 neighborhoods. Neighborhoods were represented by the population weighted centroids of each ZIP-5. Kulldorff’s retrospective space permutation method identifies the most significant and likely cluster in a study area, which is called the primary cluster [29]. The test also provides evidence for the presence of secondary non-overlapping clusters with significantly large likelihood ratios compared to the primary cluster. As the disease cases in our data set followed a Poisson distribution, we chose a discrete Poisson probability model for the clustering analysis. After conducting a preliminary test, the spatial and temporal scanning windows were defined to include a minimum of 0.5% and a maximum of 10% of the population at risk, in order to ensure a cluster size amenable to public health interventions.

In the current study, disease risks across various parts of Germany were compared. Thus, a conditional scan statistic was applied. Given the observed total number of cases (*C*), the spatial scan statistic (*S*) is the maximum likelihood ratio over all possible circles (*Z*), as calculated with the equation (Eq. 6):

6 $$S=\frac{\mathit{\max}_ZL(Z)}{L_0}={\mathit{\max}}_Z\ \frac{L(Z)}{L_0}$$

where *L(Z)* is the likelihood of observing the number of cases *c*_*z*_ within circle *Z*, given the total number of cases *C*, the population within *Z* (*n*_*z*_), and the total population *N*. The denominator *L*_*0*_ represents the likelihood function under the null hypothesis.

Clusters were tested for significance using 999 Monte Carlo simulations. For each simulation the maximum likelihood ratio statistic was calculated. Clusters with a *p*-value < 0.01 were considered significant high-risk clusters. The neighborhoods within the significant high-risk clusters were identified as high-risk neighborhoods. The relative risk (RR) [29, 30] of each oral disease for a cluster was calculated using the ratio of observed to expected cases, comparing the risk within a cluster to that outside the cluster. Each possible cluster was thus assigned an RR-value for each oral disease as defined in Eq.7:

7 $$RR=\frac{o/e}{\left(O-o\right)/\left(O-e\right)}$$

where *o* is the number of cases in the cluster, and *e* is the expected number of cases in the cluster, *O* is the total number of cases in the dataset.

### Assessing social determinants of health via machine learning algorithms

#### Selection of social determinants of health

To assess the impact of social determinants on health, all ZIP-5 neighborhoods were enriched via a data linkage process to include key independent variables such as socioeconomic status, burden of oral diseases, oral care accessibility, attitudes and beliefs, population structure, and urbanity. The predicted outcome in our study was the classification of a ZIP-5 code as belonging to a high-risk cluster. Data on socioeconomic status, population structure and urbanity were purchased from the company Panadress, data on attitudes and beliefs from the company Nexiga, and for oral care from the company Adressendiscount. For each variable, data processing was operationalized for consistency and reproducibility.

##### Socioeconomic status

For socioeconomic status, three dimensions, i.e. education, occupation, and income, were assessed according to the weights proposed by Kroll et al. [31]. For each dimension, a higher score indicated a higher socioeconomic status, whereas a lower score indicated a lower status. Data were z-standardized for comparison before weighting and dimension calculation. The dimension “education” was constructed from two variables: the percentage of inhabitants who did not complete their schooling, weighted by − 0.33, and the percentage of inhabitants with a university degree, weighted by + 0.66. The dimension “occupation” was scored using the unemployment rate (weight = − 0.61), disposable purchasing power (weight = + 0.27), and employment quota (weight = + 0.5). The dimension “income” was calculated using debtor quota (weight = − 0.41) and net household income (weight = + 0.52).

##### Attitudes and beliefs

We used the percentage of inhabitants within the four most conservative Sinus-Milieus® (©2021 Nexiga GmbH), i.e. “conservative upscale”, “nostalgic middle-class”, “precarious”, and “traditional”, to assess the attitudes and beliefs of each neighborhood.

##### Population structure and urbanity

The number of inhabitants per household and the number of inhabitants per square kilometer were used to describe population structure and urbanity, respectively.

##### Oral care accessibility

Oral care was operationalized by calculating the number of dentists per 1000 inhabitants.

##### Burden of Oral diseases

Finally, for each oral disease, the burden of oral diseases was calculated from the mean incidence rates of the other three oral diseases investigated in the study.

#### Machine learning modeling

After cleaning and preprocessing, the data was used to train and test 5 different models based on supervised machine learning algorithms, namely Logistic Regression (LR), Decision Tree (DT), Random Forest (RF), Support Vector Machines (SVM), and Neural Network (NN). For each oral disease, we selected the algorithm with the highest performance for primary model generation to accurately detect high-risk neighborhoods (based on ZIP-5), as well as test, via permutation, the importance of each independent variable assessed above in explaining disease risk.

With logistic regression (LR), we calculated the probability that the output variable (primary high-risk neighbourhood for a specific disease yes/no) belonged to the appropriate category based on neighborhood socioeconomic and other variables. A probability > 0.5 was classified as “yes” and a probability of ≤0.5 as “no”.

Decision Tree (DT) allows each node to weigh possible actions against one another based on their benefits, costs, and probabilities. A DT generally starts with a single node that branches out into several possible outcomes. Each outcome leads to additional nodes that in turn branch off into other instances, generating a tree-like shaped map or flowchart of all possible outcomes of a series of related choices. DT’s target is to maximize information gain [32].

As the name implies, the random forest (RF) algorithm generates a “forest” by assembling many decision trees. It is a supervised classification algorithm and an attractive classifier due to its high execution speed [33]. The predictions of each component tree are averaged, enabling better predictive accuracy than with a single decision tree. In general, the more trees in the forest, the more robust the forest looks.

Support Vector Machines (SVMs) are a frequently used machine learning algorithm for classification problems. In our dataset, the number of instances (ZIP-5 neighborhoods) was larger than the number of features. Therefore, we applied the non-linear Radial Basis Function (RBF) Kernel (11) to transform the non-linear problem into a linear one in a higher-dimensional space. To optimize model performance, a tuning grid was used to best determine the influence of single training points (gamma) and margin classification (cost), and to avoid overfitting.

Artificial neural networks (NNs) are deep-learning models used to achieve high accuracy in larger datasets. We applied a Feed-Forward Neural Network [34] to address non-linearity in the data and perform binary classification. The Feed-Forward Neural Network consists of an input layer, one or more hidden layers, and an output layer, and operates by accepting multiple inputs, each with its own particular weight, that are fed forward in a single direction. The network was run in machine mode to return a class label probability with one hidden layer and 5 or 6 neurons.

For model training, testing and evaluation, the datasets for each oral disease were randomly partitioned into 10 equal parts. Eight parts (80%) were used as the training dataset, and the remaining 20% served as the test data. The parameters of each model were optimized during the training process to better fit the data. Due to the high imbalance between positive and negative values for each oral disease, the Random Over Sampling Examples (ROSE) method was applied to balance the training data set for classification modeling. This approach generates artificial data based on sampling and smoothed bootstrapping methods [35], which can avoid high accuracy but low precision in predicting negative ZIP-5 neighborhoods. The models were trained to achieve both high accuracy and high precision in detecting variables important for each primary oral disease cluster.

We evaluated the performance of the various trained models using the test data. Moreover, the efficiency of all machine learning classification models was validated using various evaluation metrics as calculated based on the confusion matrix given below.

**Table:** 

Empty Cell	Predicted No (0)	Predicted Yes (1)
Actual No (0)	TN	FP
Actual Yes (1)	FN	TP

where, FP = False Positive, FN = False Negative, TN = True Negative, and TP = True Positive. Eqs. 8.1 to 8.7 were used to calculate various performance measures for each classification method:

8.1 $$\textrm{Accuracy}=\frac{\left(\textrm{TP}+\textrm{TN}\right)}{\left( TP+ TN+ FN+ FP\right)}$$

8.2 $$\textrm{Specificity}=\frac{\left(\textrm{TN}\right)}{\left( TN+ FP\right)}$$

8.3 $$\textrm{Precision}=\frac{\left(\textrm{TP}\right)}{\left( TP+ FP\right)}$$

8.4 $$\textrm{Recall}=\frac{\left(\textrm{TP}\right)}{\left( TP+ FN\right)}$$

8.5 $$\textrm{NPV}=\frac{\left(\textrm{TN}\right)}{\left( TN+ FN\right)}$$

8.6 $$\textrm{PPV}=\frac{\left(\textrm{TP}\right)}{\left( TP+ FP\right)}$$

8.7 $$\textrm{F}1\ \textrm{Score}=\frac{2\ast \left(\textrm{Precision}\ast \textrm{Recall}\right)}{\left( Precision+ Recall\right)}$$

We combined both accuracy and F1 score metrics into a single index for selecting the primary analytic model for each oral disease. Accuracy highlights the performance of the classifier and is calculated as the proportion of true positive and true negative events to the sum of all observations. The F1 score represents the harmonic average of precision (Eq. 8.3) and recall (Eq. 8.4). Receiver Operating Characteristic (ROC) curves were plotted and the area under the ROC curve (AUC) was calculated for each model as a metric of the performance of each classifier. Moreover, precision and recall metrics were used for further model evaluation. The precision (or sensitivity) determines the ability of the model to correctly detect high-risk neighborhoods based on ZIP-5, whereas recall measures the ability to find all high-risk neighborhoods. Precision-Recall (PR) curves were plotted, to address the high imbalance of the dataset [36]. Specificity, negative predictive values (NPV) and positive predictive values (PPV) were used as secondary affirmation of model selection, with specificity measuring the ability of the model to determine the non-risk neighborhoods correctly, PPV representing the likelihood of a neighborhood being truly at high-risk in case of a positive result, and NPV indicating the likelihood of a neighborhood being truly not at high-risk in case of a negative result.

Finally, an important aspect of machine learning is understanding which variables have the greatest impact on the predicted outcome. Many machine learning algorithms incorporate their own unique methods of quantifying the relative impact of each variable (i.e. coefficients for linear models, impurity for tree-based models), while others (e.g. support vector machines) do not, making it difficult to compare variable importance across multiple models. To overcome this, we used a permutation-based method that is model agnostic and therefore independent of the algorithm used. This methodology involves removing the effect of a variable through random reshuffling of the data [37] and comparing the performance of the initial model with the modified one. For each full model, the root mean squared error (RMSE) loss function was computed as shown in Eq. 9:

9 $${L}^0=\varLambda \left(\underset{-}{\hat{\textrm{y}}},\underset{-}{X},\underset{-}{y}\right)$$

where *L*^0^ represents the value of the loss function for the original data, $\varLambda \left(\underset{-}{\hat{y}},\underset{-}{X},\underset{-}{y}\right)$ represents the loss function that quantifies goodness-of-fit of the model *f()* based on log-likelihood, $\underset{-}{X}$ is the matrix with the observed values of the explanatory variables for all observations in rows, $\underset{-}{y}$ refers to the column vector of the observed values for the dependent variable *Y*, and $\underset{-}{\hat{y}}$ denotes the corresponding vector of predictions for $\underset{-}{y}$ for model *f()*.

For each explanatory variable *X*_*j*_*,* the following steps were repeated in each model. A matrix ${\underset{-}{X}}^{\ast j}$ was created by permuting the vector of observed values of *X*_*j*_ 10 times. Then the model predictions ${\underset{-}{\hat{y}}}^{\ast j}$ were computed based on the modified data ${\underset{-}{X}}^{\ast j}$ and the loss function of the modified data was calculated (Eq. 10):

10 $${L}^{\ast j}=\varLambda \left({\underset{-}{\hat{\textrm{y}}}}^{\ast j},{\underset{-}{X}}^{\ast j},\underset{-}{y}\right)$$

where *L*^∗*j*^ represents the value of the loss function.

Finally, the importance of *X*_*j*_ was quantified, calculating ${vip}_{Diff}^j={L}^{\ast j}-{L}^0$. The variable importance was recorded and presented graphically for better model comparison.

**Fig. 1:**
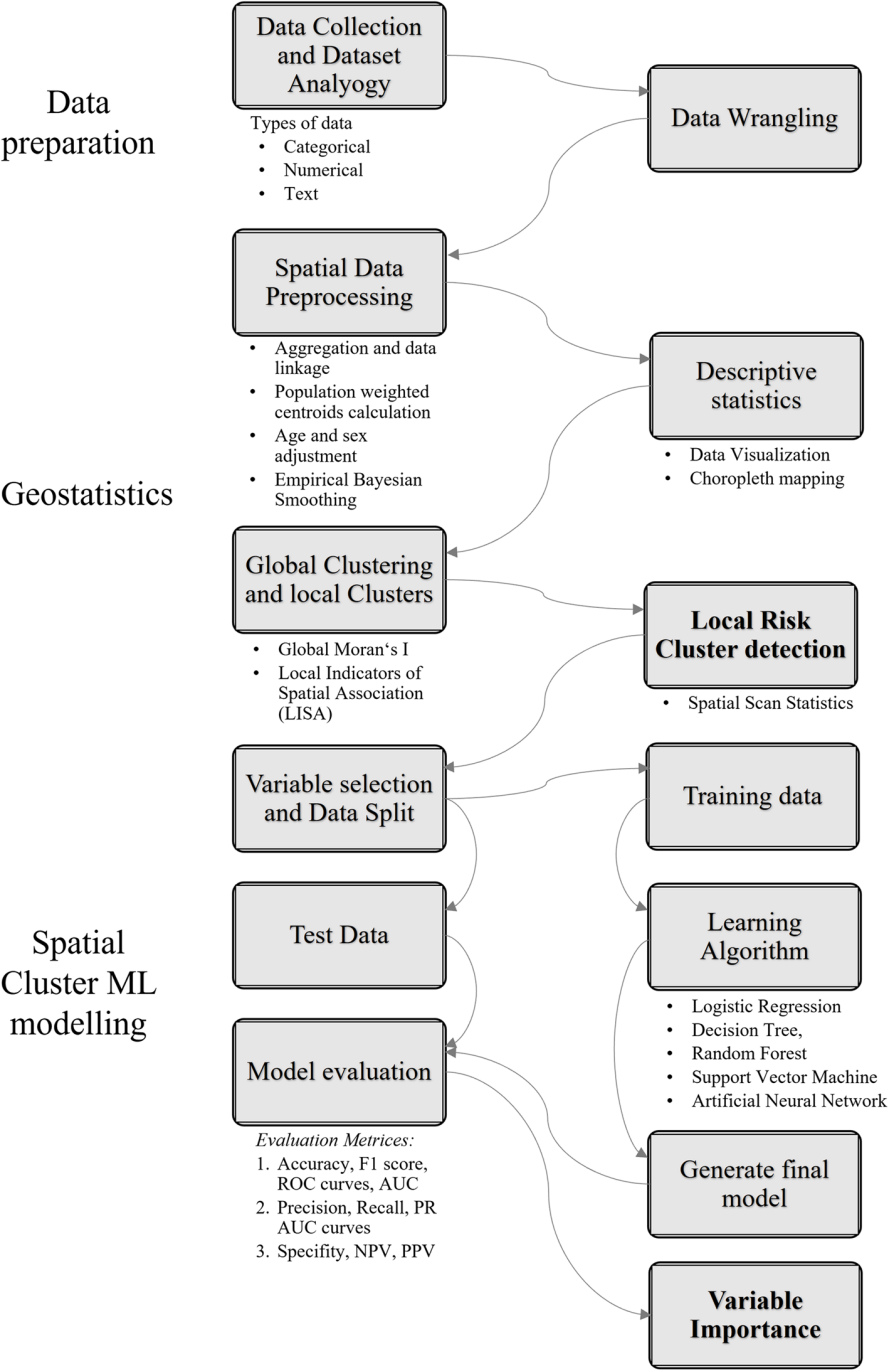
Flowchart of the study methodology to detect local oral disease risk clusters and detect neighborhood-based variables determining the clusters

The study methodology is presented in Fig. 1. Study findings are reported in accordance with the using Strengthening the Reporting of Observational Studies in Epidemiology (STROBE) [38] and REporting of studies Conducted Observational Routinely-collected health Data (RECORD) recommendations [39]. We used R (version 4.2.1) to perform statistical analyses and ArcGIS Pro 3.0 for mapping.

## Results

### Incidence rates of oral diseases

We analyzed anonymized data from 4.5 million privately insured and self-pay individuals who received dental treatment between 2016 and 2021 in Germany. Of the study population, 52.7% were female and 28% were identified as self-payers. The mean age was 46.5 years (SD = 21.6). Table 1 lists the incidence rates of the four oral diseases analyzed in this study. We calculated an incidence rate of 24.5 new cases per year per 1000 inhabitants for periodontitis, 22.0 new cases for severe caries, 44.8 cases for irreversible pulpitis, and 19.6 cases for tooth loss. Adjusting the raw rates for age and sex resulted in lower incidence rates for each disease, i.e. 21.2 for periodontitis, 15.6 for severe caries, 35.9 for irreversible pulpitis and 11.0 for tooth loss.

**Table 1:** Incidence rates of selected oral diseases of privately insured and self-payer persons 2016–2021 ^1^Adjusted for age and gender; ^2^exact Poisson 95%-CI

Oral disease	Cases	Controls	Total person-years at risk	Incidence rate raw per 1000 (95%-CI^2^)	Incidence rate adj.^1^ per 1000 (95%-CI^2^)
Periodontitis	221,548	4,241,453	9,034,205	24.5 (24.4–24.6)	21.2 (21.1–21.3)
Caries (severe)	199,367	4,263,634	9,059,976.5	22.0 (21.9–22.1)	15.6 (15.6–15.7)
Irreversible Pulpitis	395,879	4,067,122	8,832,383.5	44.8 (44.7–45.0)	35.9 (35.7–36.0)
Tooth Loss	180,494	4,282,506	9,206,381	19.6 (19.5–19.7)	11.0 (10.9–11.0)

**Fig. 2:**
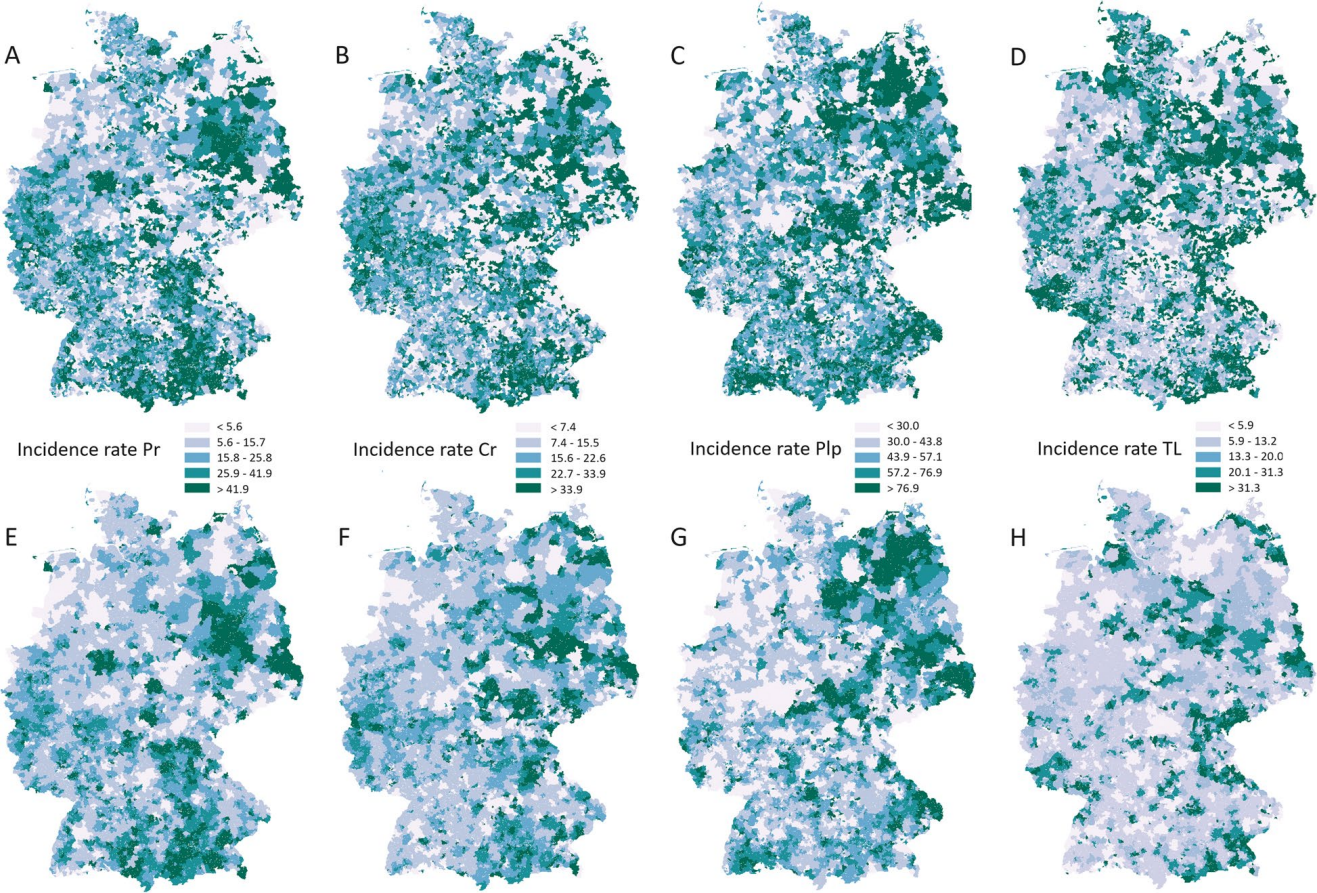
Raw (A-D) and smoothed (E-H) incidence rates of oral diseases 2016–2021 among privately insured and self-payer patients in Germany. A & E: periodontitis = Pr, B & F: caries (severe) = Cr, C & G: irreversible pulpitis = Plp, D & H: tooth loss = TL

The spatial distribution of raw and smoothed incidence rates for each oral disease is shown in Fig. 2. For periodontitis, higher incidence rates were recorded in Western Brandenburg/Saxony-Anhalt, Bavaria, and Eastern North Rhine Westphalia. The maximum smoothed incidence rate (max = 429) was observed in ZIP-5 = 17493, Mecklenburg-Western Pomerania. For severe caries, incidence rates were among the highest in Western Brandenburg/Saxony-Anhalt and Thuringia, with the maximum (max = 57) detected in ZIP-5 = 72189, Baden Wuerttemberg. In all east German states (Mecklenburg-Western Pomerania, Saxony-Anhalt, Brandenburg, Berlin, Saxony and Thuringia), higher incidence rates for irreversible periodontitis could be seen, with the highest rate (max = 580) detected in ZIP-5 = 02733, Saxony. Compared to the other oral diseases, ZIP-5 neighborhoods with higher incidence rates for tooth loss were less likely to be surrounded by other neighborhoods with similarly high incidence rates. With the exception of Berlin and Bremen, incidence rates in the highest category (> 31.3) were seen in all states, with the highest incidence rate (max = 398) recorded in ZIP5 = 09548, Saxony. For each oral disease, there was more than one ZIP-5 neighborhood with an incidence rate of min = 0.

### Spatial risk cluster analysis

#### Global spatial autocorrelation

**Fig. 3:**
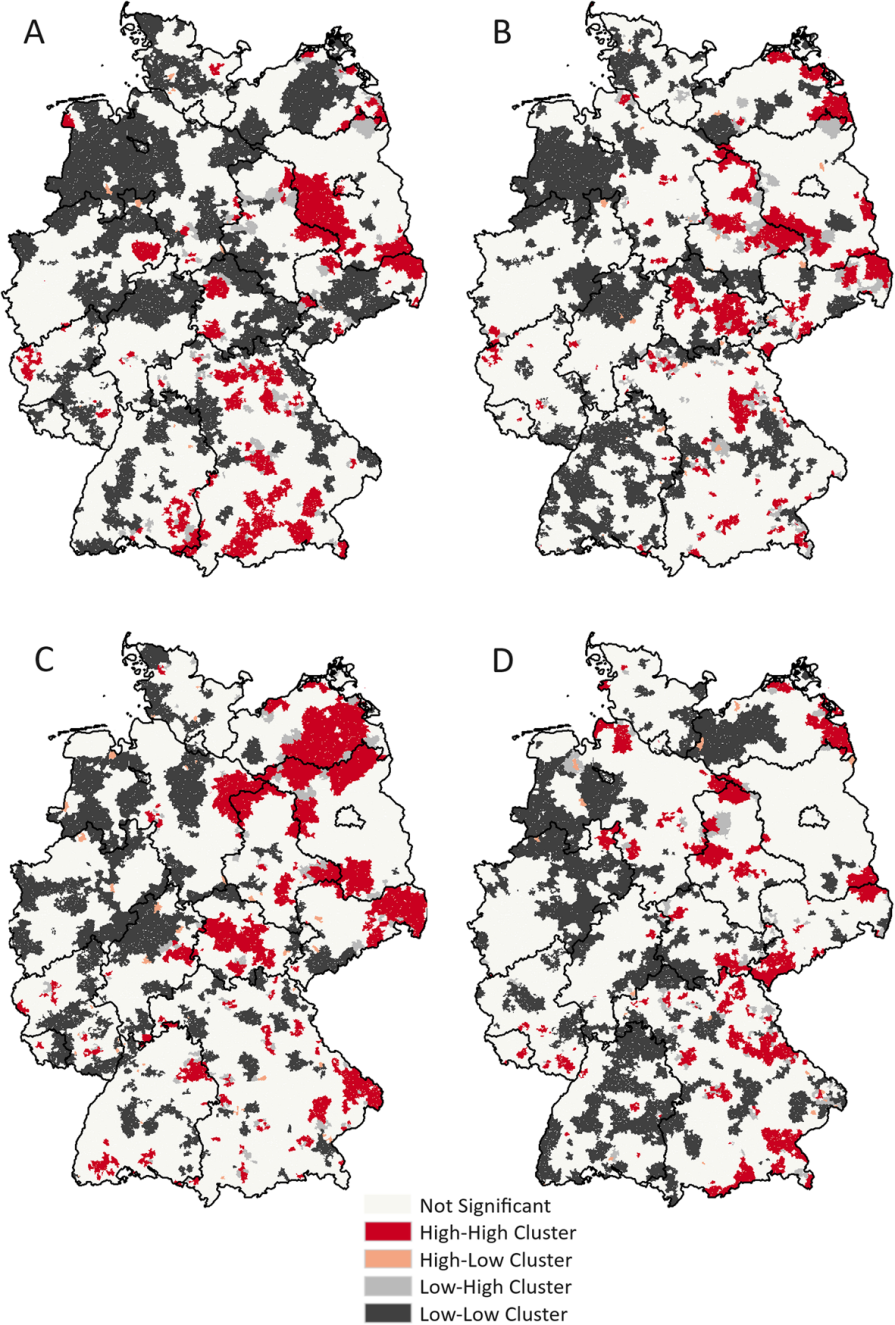
Results of the Local Moran’s *I* Test (LISA) for the four oral diseases. A: periodontitis, B: caries (severe), C: irreversible pulpitis, D: tooth loss

Before implementing the spatial cluster analyses, we examined the global Moran’s *I* statistic to evaluate the presence of spatial autocorrelation in the study area (Fig. 3). The outcome variables (incidence rates of periodontitis, severe caries, irreversible pulpitis and tooth loss) demonstrated a positive spatial autocorrelation suggesting a strong clustering pattern with a statistically significant Moran’s *I* value for all oral diseases. For periodontitis we detected *I* = 0.47 (*p*-value < 0.001, *z*-value = 68.6), for severe caries *I* = 0.25 (*p*-value < 0.001, *z*-value = 37.4), for irreversible pulpitis *I* = 0.39 (*p*-value < 0.001, *z*-value = 58.1), and for tooth loss *I* = 0.23 (*p*-value < 0.001, *z*-value = 34.6). The results indicate that the distribution of the incidence rates of each oral disease had a significant positive correlation with the incidence rate of the nearest neighborhoods during the study period. Comparing the standardized *z*-value, periodontitis and tooth loss showed the highest and lowest tendencies towards clustering, respectively, among all oral diseases.

The results of the Moran scatterplots (Fig. A1) showed a clear tendency towards classifying spatial autocorrelation into four types. Although this classification as such does not imply significance, it points to the presence of high incidence rate clusters for each disease, as evidenced by several cases of positive spatial association in the upper right quadrant. However, the presence of deviant associations in the upper left, as well as the deviation of points from the linear regression line suggest the need for additional measures besides spatial autocorrelation measures, and highlight the limitations of using a single global measure for the analysis of spatial association in the data [40].

#### Local Moran’s I

**Fig. 4:**
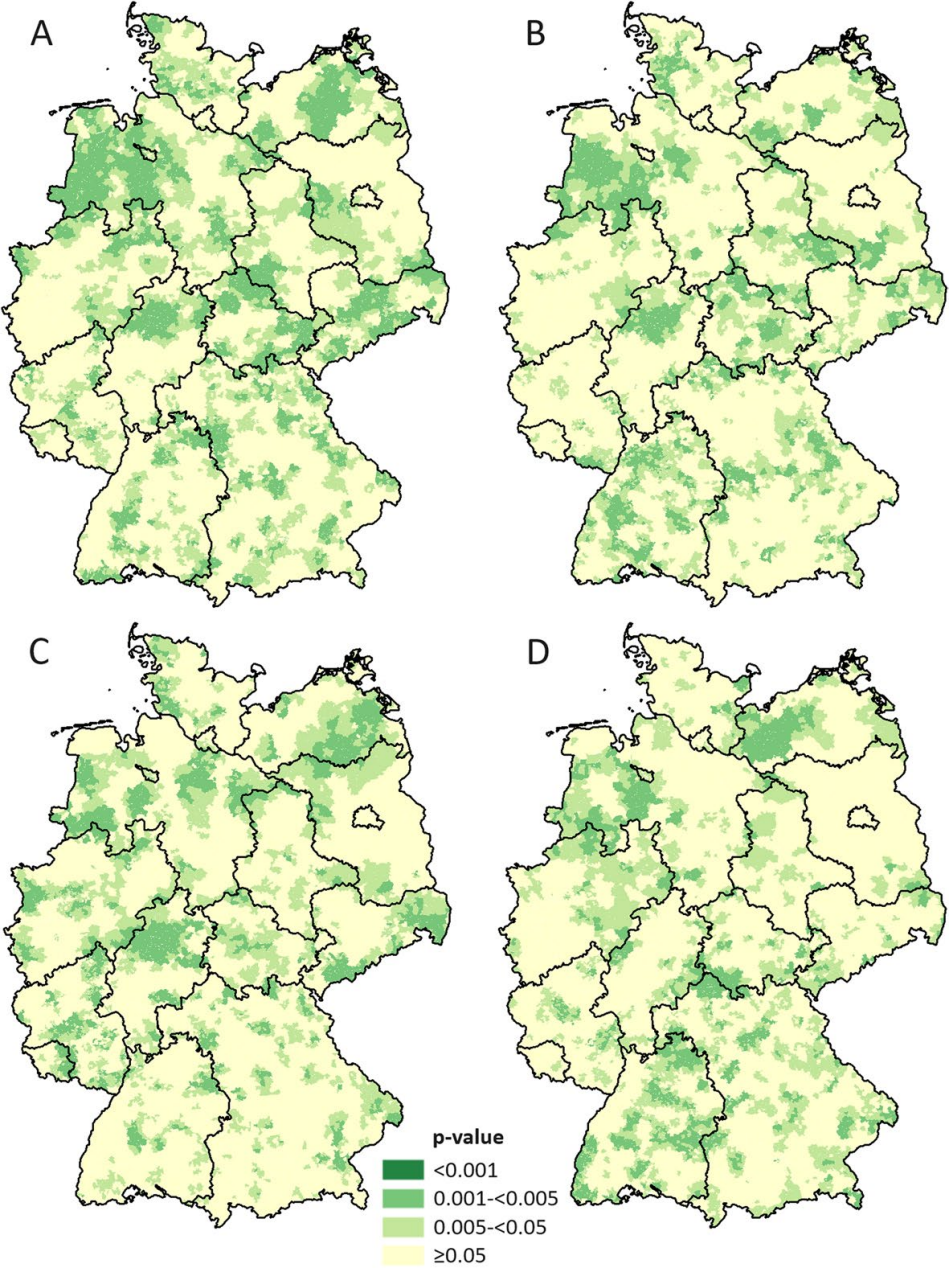
*P*-values of the Local Moran’s *I* Test (LISA) for the four oral diseases. A: periodontitis, B: caries (severe), C: irreversible pulpitis, D: tooth loss

To further explore the relationship between global and local spatial autocorrelation, we extended our analyses using Local Moran’s Indicators of spatial associations (LISA) to identify oral disease clusters with a significance level of 5% among the German ZIP-5 neighborhoods. The results revealed distinct, statistically significant high-high clustering or ‘hotspots’ in the eastern part of Germany for all oral diseases (Figs. 3 and 4). For periodontitis, major hotspots were identified in an area west of Berlin, encompassing regions of Brandenburg and Saxony-Anhalt, at the border between Brandenburg and Saxony, and in areas of Bavaria and Baden-Wuerttemberg. Smaller hotspots could be detected in all states, with the exception of Saarland and Hamburg. For severe caries the largest cluster could be detected along the border between Brandenburg and Saxony-Anhalt, with smaller hotspots in all other states, except for Berlin, Hamburg and Saarland. Larger clusters were detected in Mecklenburg-Western Pomerania, Saxony-Anhalt, Brandenburg, Saxony, Thuringia, and Bavaria for irreversible pulpitis, and in Saxony-Anhalt, Thuringia, Saxony, and Bavaria for tooth loss. For each disease, there was no neighborhood detected that had a *p*-value of > 0.001, indicating the need for additional methods to detect local clusters. Nevertheless, the analysis enabled the identification of significant local clusters, allowing the application of more targeted approaches, which helped to address the severe shortcomings of the Local Moran’s *I* methodology, such as the multiple testing problem and inappropriate weighting matrices.

#### Spatial scan statistics

**Fig. 5:**
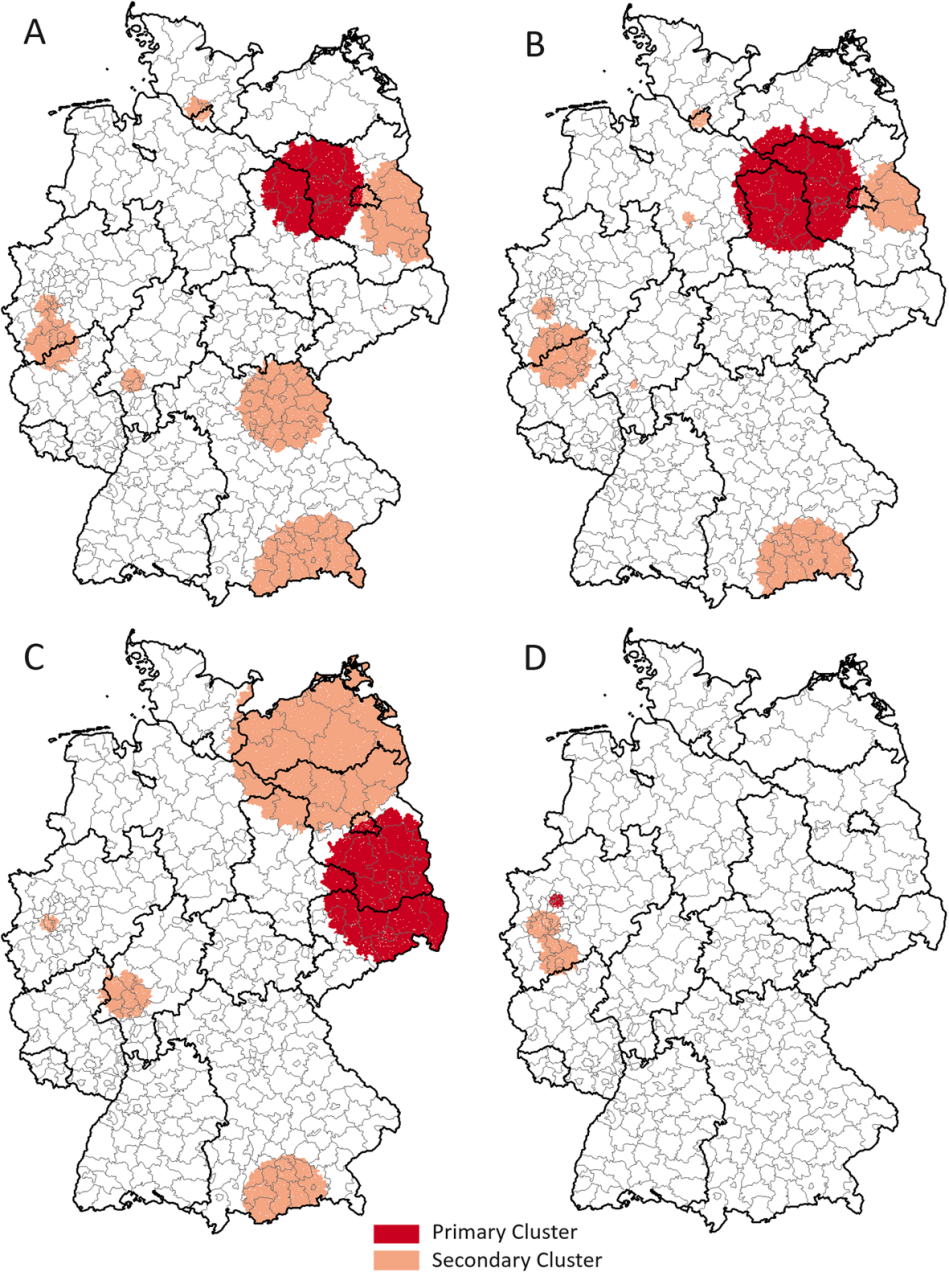
Significant primary and secondary risk clusters for incidence rates of oral diseases in Germany 2016–2021 detected by spatial scan statistic. A: periodontitis, B: caries (severe), C: irreversible pulpitis, D: tooth loss

Using spatial scanning methods, we identified spatial clusters of each of the four oral diseases in different parts of Germany (Fig. 5). Table 2 presents space-time cluster characteristics for each oral disease, including Cluster ID, total number of neighborhoods, observed and expected number of cases, incidence rate in and outside the cluster, *p*-values, and the relative risk rates for each primary cluster. High-risk clusters varied in terms of size, magnitude of relative risk, and total number of neighborhoods affected. Moreover, significant secondary clusters could be identified for each oral disease investigated: 7 each for periodontitis and severe caries, 4 for irreversible pulpitis, and two for tooth loss, respectively. The highest relative risk (RR = 2.7) was recorded for periodontitis in the eastern part of Germany, spanning the states Saxony-Anhalt, Brandenburg, and Berlin. The primary cluster for severe caries overlapped with the primary cluster for periodontitis and additionally extended to parts of Mecklenburg-Western Pomerania and Lower Saxony with a RR = 1.6. The irreversible pulpitis primary cluster was located in Eastern Germany, in the states of Saxony, Brandenburg, Berlin, and Saxony-Anhalt (RR = 1.3). The primary cluster for tooth loss was located in western Germany, in the state of North Rhine-Westphalia, in the northern Ruhr area.

**Table 2:** Description and relative risks (RR) of primary risk clusters of oral diseases detected in spatial scan statistics

Primary Cluster	No. of ZIP-5 neigh-bor-hoods	Obs. no. of cases	Exp. no. of cases	Incidence rate in cluster per 1000	Incidence rate outside cluster per 1000	RR (95%-CI)	*p*-value
Periodontitis	370	13,348	4996	47.6	20.5	2.7 (2.6–2.8)	< 0.001
Caries (severe)	288	7240	4560	24.7	16.2	1.6 (1.5–1.6)	< 0.001
Irreversible Pulpitis	441	18,549	14,074	49.1	38.0	1.3 (1.3–1.3)	< 0.001
Tooth Loss	50	2745	1833	17.4	12.6	1.5 (1.4–1.6)	< 0.001

### Identifying important variables for primary cluster detection

To develop models for each of the four oral diseases, ZIP-5 neighborhood data was enriched to include key independent variables of social determinants of health. We applied five different machine learning algorithms to generate models to predict ZIP-5 neighborhood primary risk clusters for each oral disease. The performance of each model was then evaluated based on several metrics, as listed for each disease in Table 3.

**Table 3:** Metrics of the five Machine Learning Models to predict ZIP-5 neighborhood primary risk clusters of the four oral diseases

	Accuracy [%]	Specificity[%]	Precision[%]	Recall[%]	NPV [%]	PPV [%]	F1 Score	AUC (ROC)
Periodontitis								
Logistic Regression	86.8	91.9	99.8	86.7	13.8	99.8	0.93	0.97
Decision Tree	87.3	73.0	99.3	87.7	12.1	99.3	0.93	0.80
Random Forest	93.5	59.5	99.0	94.2	19.3	99.0	0.97	0.77
Support Vector Machine	92.1	75.7	99.4	92.5	18.9	99.4	0.96	0.95
Neural Network	88.2	78.4	99.4	88.5	13.6	99.4	0.94	0.95
Caries (severe)								
Logistic Regression	83.2	75.9	98.9	83.5	14.5	98.9	0.91	0.85
Decision Tree	78.4	63.8	98.3	78.9	10.0	98.3	0.88	0.71
Random Forest	88.0	62.1	98.5	89.0	17.1	98.5	0.93	0.76
Support Vector Machine	89.2	62.1	98.5	90.2	18.9	98.5	0.94	0.86
Neural Network	88.1	62.1	98.5	89.1	17.3	98.5	0.94	0.80
Irreversible Pulpitis								
Logistic Regression	88.6	96.6	99.8	88.2	31.7	99.8	0.94	0.95
Decision Tree	87.8	93.2	99.6	87.5	29.8	99.6	0.93	0.90
Random Forest	90.1	86.4	99.1	90.3	33.6	99.1	0.95	0.88
Support Vector Machine	89.8	87.5	99.2	90.0	33.2	99.2	0.94	0.95
Neural Network	91.5	70.5	98.2	92.7	35.4	98.2	0.95	0.91
Tooth Loss								
Logistic Regression	90.5	90.0	99.9	90.5	5.5	99.9	0.95	0.96
Decision Tree	82.6	90.0	99.9	82.6	3.1	99.9	0.90	0.86
Random Forest	86.2	100.0	99.9	86.1	4.3	99.9	0.93	0.93
Support Vector Machine	84.8	100.0	99.9	84.7	3.9	99.9	0.92	0.98
Neural Network	73.6	100.0	99.9	73.5	2.3	99.9	0.85	0.95

For periodontitis, the RF model showed the highest values for accuracy and F1 score, but returned the lowest scores for precision and AUC (ROC) (Fig. A2) among all models. In comparison, the LR model had the highest scores for precision and AUC (ROC). For severe caries, SVM had the highest accuracy and highest F1 score (together with NN). LR showed the highest precision, and SVM the highest AUC (ROC). NN showed the best performance regarding accuracy and F1 score (together with RF) for irreversible pulpitis. LR had the highest precision, and LR and SVM the highest AUC (ROC). PR curves showed RF and DT to perform better than the other models. Regarding tooth loss LR had the highest accuracy and was the only model to have an accuracy above 90%. LR had the highest F1 score. No difference between the models were detected regarding precision, while SVM had the highest AUC (ROC) score. Generally, the models for tooth loss had lower PR curve scores compared to all other diseases (Fig. A3). PR curves showed SVM to perform better than all other models for periodontitis, severe caries and tooth loss. DT showed the best performance for irreversible pulpitis. Based on these metrics, we determined SVM to be the best performing model for periodontitis and severe caries, whereas NN and LR performed best for irreversible pulpitis and tooth loss, respectively.

Having identified the best-performing model for each disease, we used a model-agnostic permutation-based method for calculating variable importance, identifying those variables with the greatest impact in detecting primary risk clusters for each disease (Fig. A4). For both periodontitis and severe caries, the best model (SVM) revealed that affiliation to the primary cluster was determined by several factors, including a rural location, low income, and a preference for traditional cultural values independent of status (Table 4). Moreover, there were tendencies towards a lower incidence of other oral diseases, and reduced access to dental health care. Additionally for severe caries, a lower education level was also an important determinant. In the best-performing model of irreversible pulpitis (NN), the primary risk cluster was associated with higher education levels, smaller household size, a rural living environment, a less-favorable occupation status, and a preference for traditional values and health behavior (i.e. as associated with the nostalgic middle-class milieu). Finally, the LR model for tooth loss, highlighted key variables to be a smaller household size, a more urban living area, lower income, and a precarious and nostalgic attitude towards health. There was also a tendency towards higher incidences of other oral diseases, as well as a less-favorable occupation status.

**Table 4:** Root-mean square error (RMSE) loss for independent variables of best performing ML models, including the direction of association, to detect primary cluster ZIP5 neighborhoods for each oral disease. Values of variables over 0.01 RSME loss are shown in bold

Variable	Periodontitis (SVM)	Caries (severe) (SVM)	Irreversible Pulpitis (NN)	Tooth Loss (LR)
Baseline	0.059	0.051	0.092	0.013
Education	0.004	0.007	**0.011**	0.002
Inhabitants per household	0.003	−0.003	**−0.022**	**−0.019**
Inhabitants per km^2^	**−0.020**	**−0.023**	**−0.012**	**0.011**
Incidence other oral diseases	−0.005	0.000	−0.001	0.003
Income	**−0.011**	**−0.016**	−0.004	**−0.010**
Occupation	−0.001	0.004	−0.008	−0.006
Conservative upscale milieu	0.009	0.009	0.000	0.005
Nostalgic middle-class milieu	0.004	0.003	**0.039**	0.009
Precarious milieu	0.004	0.003	0.002	**0.018**
Traditional milieu	**0.019**	**0.017**	0.008	−0.004
Dentists per 1000 inhabitants	−0.001	− 0.001	0.000	0.003

## Discussion

### Descriptive statistics of incidence rates

Our study analyzes the incidence rates of the oral diseases periodontitis, severe caries, irreversible pulpitis and tooth loss over a 6-year period in Germany, based on data from ~ 4.5 million privately insured and self-payers, and thus represents one of the largest collections of data on oral diseases to date.

Validating our analyses against independent data sources is important to verify our results. However, this is confounded by the scarcity of studies reporting on the incidence rates of oral diseases. In our review of the literature, we found only a limited number of studies focusing on incidence rates compared to those focusing on prevalence, an issue that is also discussed in a systematic review by Kassebaum et al. [41]. Additionally, in our study, the calculated incidence rates are based on secondary claims data, making a direct comparison with results from the literature more difficult. Most studies rely on individual assessments of diseases by trained professionals for disease detection, which is considered to be more accurate than those based on claims data. However, such studies often do not include a large number of participants, thereby limiting their utility for external validation of results. For example, a Japanese study reported an incidence rate of 19.0 for periodontitis in a 6-year follow-up study of older adults [42]. While this rate is similar to the incidence rate we report here of 21.2, also considering the socioeconomic and health care similarities between Japan and Germany, the Japanese study only included 374 individuals, thereby limiting its generalizability. Other studies include a 24-year follow-up study of self-reported periodontitis in ~ 35,000 male health professionals between the ages of 40 and 75 years in the USA, which reported an incidence rate of 5.3, lower than what we detected here. However, we assume that health professionals are more likely to seek attention for health and dental issues early, and a self-report is a weak measure of actual oral disease, especially regarding periodontitis. In one review, an age-standardized incidence rate of 0.7 per 1000 persons per year for severe periodontitis in Western Europe was calculated [41], but included only persons with a gingival pocket depth ≥ 6 mm. There are also studies reporting higher incidence rates, including a 4-year follow-up study that assessed gingival recession in 402 adults ≥35 years in Brazil, which reported an incidence rate of 359 per 1000 per year [43]. Differences in oral health care services and dental health status between Germany and Brazil, as well as the inclusion of younger individuals in our study, could account for the differences in reported incidence rates. In terms of study design, study population characteristics, and sample size, the recent study of Rädel et al. [44] was most similar to our study here, and thereby allows a meaningful comparison with our results. Rädel et al. used claims data of ~ 9 Mio. publicly insured persons in Germany. Diseases were defined based on figures from the dental fee schedule for publicly insured patients (BEMA). Of note, this study calculated an incidence rate of 19 for the year 2019, corroborating the results of our study.

There is limited research on the incidence rates of severe caries, including direct or indirect pulp capping. A study from Mexico found a maximum incidence rate of 31.5 for new lesions in 88 schoolchildren over a 5-year period. However, the exact dates of occurrence of the lesions were not reported [45]. In this present study, we calculated a general caries incidence rate of 194.1, which also includes smaller lesions not affecting the pulp. Considering our results, other studies generally show comparable incidence rates. A systematic review of data from 291,170 people from 37 countries worldwide found an age-standardized incidence rate of 272.6 for 2010. Two studies reported a higher incidence rate: a 3-year follow-up study of individuals > 50 years of age in Canada reported an incidence rate of 570 [46], and a 2-year follow-up study from Florida, USA, reported an incidence rate of 670 [47]. The study by Rädel et al. [44] calculated an incidence rate of 261, which is comparable to the incidence rate we calculated here for caries in general. However, there remains a large gap in our knowledge regarding the incidence rates of severe caries.

The incidence rates reported for irreversible pulpitis vary in the literature. Rädel et al. reported an incidence rate of 52 in Germany for 2019, comparable to our incidence rate (35.9) here. A study in Brazil showed an incidence rate of 49 [48] in a 4-year follow-up assessment of root caries in adults. Two separate studies, reported similar incidence rates of 30 and 31.5, respectively, for retreatment and apicoectomy in endodontic procedures in the USA [49], and endodontic interappointment emergencies (EIE) with necrotic pulp and retreatment in Singapore [50]. A 10-year follow-up US study reported an incidence rate of 105.9 for root canal therapy [51], whereas a 1-year follow-up study of dental surgery patients calculated an incidence rate of 78 for root canal filling, necrotic pulp, or irreversibly pulpitis [52]. These higher rates are consistent with the focus on specific high-risk cohorts in these studies.

Very few studies are available regarding incidence rates for tooth loss, with most focusing primarily on edentulism [53]. A 6-year follow-up study of older adults in Japan calculated an incidence rate of 8.2 [42]. Rädel et al. reported an incidence rate of 82 for tooth loss in Germany in 2019 [44]. We report a lower incidence rate here. A possible explanation for this is the likelihood that privately insured individuals and payers have better access to dental care [54]. This line of reasoning is supported by the fact that tooth extraction is considered to be the last treatment option for an oral disease.

In summary, our first null hypotheses could be rejected. Our study is comparable to other studies, especially for incidence rates of periodontitis and pulpitis, and can be regarded as representative for oral diseases in Germany. This also means that health data from private companies, in this case a private claims service provider, can be used to estimate disease burden and care needs in Germany [55–57]. In order to harness the full potential of health data, the findings from multiple data sources would have to be analyzed collectively.

### Spatial clusters of oral diseases

Maps help us visualize complicated geographic information. They help identify patterns in data that are otherwise abstract to the reader. The first step in taking public health action should be to map patient needs [6]. This can be accomplished by using claims data, as in our study, or clinical data. Predicting the at-risk population, as well as their current and future specific needs, will aid in the design of interventions specific for the target population [58].

The results of our analysis of oral disease incidence show non-random spatial distribution patterns in Germany. The spatial scan statistics could reveal overlapping clusters for periodontitis and severe caries in eastern Germany. The pathogeneses of these two diseases are different, as dental caries affects the enamel and dentin, whereas periodontitis that affects the gums. The data suggests that overall oral health in this region may be compromised in a variety of ways. Thus, cluster analysis can be used to identify potential areas of risk so that preventive measures can be taken. The aim should be to provide the right oral care at the right location.

Our results are in line with recent studies that found elevated risk of caries in specific areas. Strömberg et al. [59] could identify clusters in the Halland region in Sweden, with smoothed relative risks of up to 2.37 for preschool children. Antunes et al. [12] and Pereira et al. [13] found that caries risk was clustered in the outskirts of Sao Paulo, Brazil. These studies highlight regions that warrant the attention and focus of public health practitioners and policymakers. However, there is a lack of studies that consider spatial patterns (such as clustering and risk clustering) for other age groups and other oral diseases. Studies have shown that oral cancer, also appears to be spatially clustered [60]. In our study, we could detect spatial clusters for periodontitis, irreversible pulpitis, and tooth loss.

There were similarities and dissimilarities in the disease clusters detected by the two different clustering approaches. In this respect, it is important to note that local Moran’s *I* does not take multiple testing problems into account and does not calculate the magnitude of the risk, whereas the scan statistic does. The specific value of spatial scan statistics could be demonstrated in this study, because local Moran’s *I* methodology failed to detect the tooth loss cluster in Western Germany. Overall, the clusters identified by the scan statistic were more concentrated and localized, but were detected over a larger region than those identified by local Moran’s *I*. With local Moran’s *I*, clusters are identified in a strictly bounded area, where the correlation is assessed between the disease rate of a certain neighborhood and those of neighboring areas as defined by the weights matrix [61]. Scan statistic may represent a more sensitive method, and compared to local Moran’s *I*, additionally provides more detail on the cluster’s characteristics, such as the number of cases, radius, and relative risks [62]. Furthermore, the exploratory nature of scan statistics helps to uncover hidden spatial structures that would otherwise remain undetected with conventional methods. For public health interventions and to ensure logical consistency of analysis results, we advocate a combined approach that first uses local Moran’s *I* to test generally for the existence of local clusters, followed by the use of scan statistics to provide an in-depth understanding of the disease clusters, risk assignments, and prioritization.

Thus, our second null hypotheses could be rejected. The spatial dimension is an important determinant of oral diseases, and oral diseases are spatially clustered in Germany.

### Importance of social determinants of health in oral disease high risk cluster neighborhoods

Geographic correlation research assesses the relationship between various socioeconomic, demographic and lifestyle factors and health outcomes, as measured on a geographic scale. Using machine learning algorithms, we aimed to generate disease models capable of detecting high-risk cluster ZIP-5 neighborhoods for each disease, and additionally identify variables significantly associated with these clusters. All models performed better than the random model, but generally, overall performance was considered to be moderate. As we aimed for high precision and accuracy in our models, the use of oversampling techniques during the training process was considered to be successful. All models, regardless of disease, had high precision scores and moderate to high accuracy scores.

For periodontitis, SVM scored high for accuracy, F1 score, precision, and AUC (ROC). The PR curve additionally showed that precision was higher at low recall values compared to all other models. Recall, NPV, and PPV scores also supported the choice of SVM as the best-performing model for periodontitis risk cluster detection. For severe caries, all models showed lower AUC (ROC) values when compared to the other diseases. Nevertheless, SVM performed consistently well in all metrics, including recall, NPV, and PPV, and was selected as the best model for variable significance detection. The overlapping clusters in both diseases may explain why the ML models showed similar results in explaining the primary clusters. In both diseases, SVM was determined to be the best-performing model.

For irreversible pulpitis, NN performed similarly to LR and SVM in terms of precision and AUC (ROC) scores, but showed the best performance in precision and F1 score. NN was therefore selected as the best model for variable significance determination. All models for tooth loss performed comparatively poorer to the other diseases, potentially due to the extreme imbalance and low number of cases in the data. Further research with different oversampling techniques may yield better results. LR was selected as the best model for determining variable importance in detecting clusters of neighborhood risk because of its accuracy, F1 score, and high AUC (ROC).

We evaluated the importance of several variables in our ML models by analyzing RMSE after permutation. Variables with the largest RMSE were considered to be the most influential for each model, as their removal resulted in significant loss of accuracy. This method enabled us to identify variables that were important for all models, multiple models, or no models, additionally allowing us to assess whether the models picked up on unique patterns in the data, or whether they used a common logic.

In our analyses of the four disease-specific models, we found the most important determinants for identifying primary clusters in ZIP-5 neighborhoods to be income, a traditional health behavior, and a higher rurality. Lower income was a significant factor in predicting primary risk clusters in periodontitis, severe caries and tooth loss. Several studies have confirmed lower socioeconomic status to be a key factor for developing an oral disease, especially caries [12, 13, 16]. Although income was not as important a factor for irreversible pulpitis as in other disease models, a poorer occupational status showed a high correlation with disease risk. Thus, our results also highlight the importance of socioeconomic status in the development of irreversible pulpitis.

The importance of health behavior is evidenced in the literature. It is well-accepted that an “unhealthy” lifestyle, which includes smoking, alcohol consumption, an unbalanced diet, stress, and missed preventive examinations, is a primary and significant risk factor for developing disease [63]. Our analyses found a traditional attitude towards healthcare to be a key variable in predicting oral disease risk that was significant in all models, regardless of status. A traditional attitude to healthcare is more oriented toward repair medicine than preventive measures, e.g. seeking dental care only in emergency situations [64]. The importance of this variable, however, may reflect a bias in the data, as only people who visited a dentist for disease-specific procedures during the study period could be included in the analyses.

All four models appear to be largely influenced by rurality (population per km^2^). However, the direction of impact was different for periodontitis, severe caries, and pulpitis, in which rurality was a positive predictor of primary clusters, than for tooth loss, wherein urbanity was identified as a negative predictor. Rurality has been validated as a predictor in other studies [12, 13] and may reflect the negative effects of poorer accessibility to dental care. This is also confirmed in our results by the trend towards lower dental care as a predictor of periodontitis and severe caries. The findings of urbanity may be of particular interest in the context oral health diseases, and underscores the need for cluster-specific interventions rather than a blanket approach which is not likely to be efficacious. That urban areas are susceptible to forming clusters has also been noted in previous studies [16]. Of note, urban areas also have socioeconomically disadvantaged populations who cannot afford or have limited access to oral health care. Further investigation is needed to better understand the differences in oral health care between urban and rural areas.

Recent studies show a link between lower levels of social support and higher prevalence of oral diseases [65]. Results for the current study found that people living in households with fewer individuals are at higher risk of being in a cluster for irreversible pulpitis or tooth loss. While this variable may not fully represent social support, it potentially reflects a lower number of social interactions in households with fewer people. There is also evidence that single status is a risk factor for mortality [66]. However, the impact of social interactions and social support in oral diseases requires further investigation, as these factors have generally not been considered in previous studies. Indeed, the socioeconomic factors contributing to oral diseases have not been fully elucidated in the literature.

Minor differences are noted in the importance of the variable “incidence of other oral diseases”. This variable is not a predictor of cluster risk in severe caries. This is potentially because caries is one of the first oral diseases that people develop. In contrast, irreversible pulpitis and tooth loss are often associated with treatment after the initial caries experience. Along this line, the significant impact of the (co-)incidence of other oral diseases to periodontitis, could be a reflection of periodontitis patients having had other oral health problems in the recent past.

In general, our third null hypotheses was rejected. Our study could show that machine learning models produced plausible results, based on performance data and with regards to social determinants of health. The varying significance of variables across clusters indicates the need for tailored (i.e. cluster-specific) health measures. Despite some similarity in the importance of variables across different clusters, each cluster is unique and must be treated as such when addressing oral public health threats. Interventions must therefore be risk group-specific, disease-specific, and patient-centered. Recognizing spatial clusters is an essential first step to successfully delivering the right care at the right location.

### Critical reception and outlook

It is important to note some methodological limitations of the study. We calculated incidence rates based on uniquely identified individuals (self-payer or privately insured), adopting a public health perspective that made assumptions about the new cases of oral disease without focusing on the severity of the disease, such as the number of affected teeth. We assumed that if a disease presents in a person, there is a need for public health intervention, as conventional prevention measures failed to prevent the disease. In our study, we exclusively included patients who received dental treatment for their respective conditions. Consequently, individuals with oral diseases who did not undergo treatment were not represented in our sample, which could potentially skew the estimation of incidence rates. Nonetheless, studies employing claims data to compute incidence rates have demonstrated the capability to yield representative outcomes [67].

The data used was not originally created to detect incidence rates, but to reimburse medical services. Individuals already suffering from an oral disease, but who did not visit a dentist during the study period were therefore not included. Therefore the actual number of cases may be underreported. Additionally, there is a potential bias of misclassification, i.e. if an incorrect procedure was provided by the medical staff. This risk is likely to be low for privately-insured persons in the study, as such reimbursements are subject to rigorous verification procedures by private health insurers. We addressed this risk by focusing on procedures clearly linked to the specific oral diseases. The use of incidence rates in this study reflects the actual demand for medical services and does not account for individuals who might need medical attention, but have not sought it. Moreover, the calculation of incidence rate using person-time assumes that the likelihood of disease remains constant over the study period, so that observing 5 persons for one year is equivalent to following one person for 5 years.

The spatial scan statistic uses a circular window to distinguish disease clusters and is unable to detect clusters irregular in shape [68]. However, on a national scale, the methodology should help to primarily detect geographic locations in need of further public health investigations. Moreover, we used a limited number of variables, selected from commercially available data at the ZIP-5 neighborhood level, to describe the clusters. Future studies should include additional explanatory variables, e.g. smoking habits, nutrition, and dental hygiene, all known to impact oral disease incidence rates [2], to enable a more comprehensive description of the factors driving disease risk.

In Germany, oral health diseases pose a significant threat to public health at all ages. Several ongoing preventative measures are adopted by the German government and local health authorities to control the spread of oral diseases. However, while studies report a decline in the number of cases [3], incidence rates of oral diseases still remain high. Spatial modeling of disease enables an understanding of the magnitude of the risk in different regions of the country. Some studies have emphasized the importance of early intervention strategies to mitigate oral disease risk [2]. Our study has several policy implications for mitigating the risk of oral diseases:

(i) it can serve as a spatial guideline for decision-makers, facilitating the formulation of mitigation strategies that focus on hotspot ZIP-5 neighborhoods;
(ii) it can provide explicit information about the spatial drivers of oral diseases, enabling policymakers to establish targeted disease surveillance measures based on the specific socioeconomic risk determinants of the neighborhoods;
(iii) it could aid in the development of plans for decreasing socioeconomic inequalities in high-risk neighborhoods;
(iv) it can identify areas where strategies to enhance public awareness and knowledge of oral diseases are needed;
(v) it may help identify areas for the development of practice-focused innovative health care solutions, treatments and therapy, as privately insured and self-payers are more likely to consume innovative health care solutions.

Oral health goals and policies should be based on high quality, up-to-date descriptive and analytical epidemiology data. This study proposes a methodology for independent and continued monitoring of oral health goals and objectives across all geographic areas, enabling comparisons over time that can be leveraged for effective and efficient policy development in oral healthcare.

## Conclusions

This study aimed to analyze the incidence of the oral diseases periodontitis, severe caries, irreversible pulpitis and tooth loss in Germany, spatially locate the demand for care, and identify and characterize high-risk clusters for targeted intervention. Our results show that private claims data can be used to highlight locations and variables relevant to oral healthcare. A network-based, data-driven approach, that includes the use of non-traditional data sets, holds great potential in supporting resource-based management in the health system. Using spatial methods to model oral disease incidence could improve current prevention and control strategies at the ZIP code level. Our study adopts multiple spatial models to garner deeper insight into the risk of oral diseases in Germany, identifying several high-risk clusters across the country in need of cluster-specific, targeted interventions for effective, patient-centered disease control. Our results also highlight socioeconomic determinants of health, such as income and occupational status, as potential underlying factors contributing to disease risk, and underscore the need to address these aspects for effective disease surveillance and control. People living in poorer socioeconomic conditions are less likely to adhere to proper prevention measures or avail of dental services. The findings of our study can inform policymakers and researchers in focusing on oral disease incidence and socioeconomic predictors to mitigate disease risk and improve oral health.

### Supplementary Information

**Additional file 1: Supplementary Figure A1.** Moran scatterplots of oral diseases in Germany. A: periodontitis, B: caries (severe), C: irreversible pulpitis, D: tooth loss.

**Additional file 2: Supplementary Figure A2.** ROC Curves for ML Classification methods. A: periodontitis, B: caries (severe), C: irreversible pulpitis, D: tooth loss.

**Additional file 3: Supplementary Figure A3.** PR AUC Curves for ML Classification methods. A: periodontitis, B: caries (severe), C: irreversible pulpitis, D: tooth loss.

**Additional file 4: Supplementary Figure A4.** Results of the permutation-based variable importance calculation based on root mean squared error (RMSE) loss for each disease, each model and each variable. The best performing model for each disease is highlighted. A: periodontitis, B: caries (severe), C: irreversible pulpitis, D: tooth loss.

**Additional file 5: Supplementary Table A1.** Hyperparameters and Functions of Machine Learning Models for periodontitis. **Supplementary Table A2.** Hyperparameters and Functions of Machine Learning Models for severe caries. **Supplementary Table A3.** Hyperparameters and Functions of Machine Learning Models for irreversible pulpitis. **Supplementary Table A4.** Hyperparameters and Functions of Machine Learning Models for tooth loss.

## Data Availability

The datasets used and analysed during the current study are available from the corresponding author on reasonable request.

## Abbreviations

RR: Relative Risk
DMFT: Decayed missing filled teeth)
ZIP-5: ZIP code 5 level
LISA: local indicator of spatial association
LR: Logistic Regression
DT: Decision Tree
RF: Random Forest
SVM: Support Vector Machines
NN: Neural Network
ROSE: Random Over Sampling Examples
ROC: Receiver Operating Characteristic
PR: Precision-Recall
NPV: Negative predictive values
PPV: Positive predictive values
RMSE: Root mean squared error
RECORD: REporting of studies Conducted using Observational Routinely-collected health Data
