# Supplementary figures and images for: Detecting high-risk neighborhoods and socioeconomic determinants for common oral diseases in Germany
Source: BMC Oral Health. 2024 Feb 9;24:205. doi: 10.1186/s12903-024-03897-4 (PMC11360568; doi:10.1186/s12903-024-03897-4)

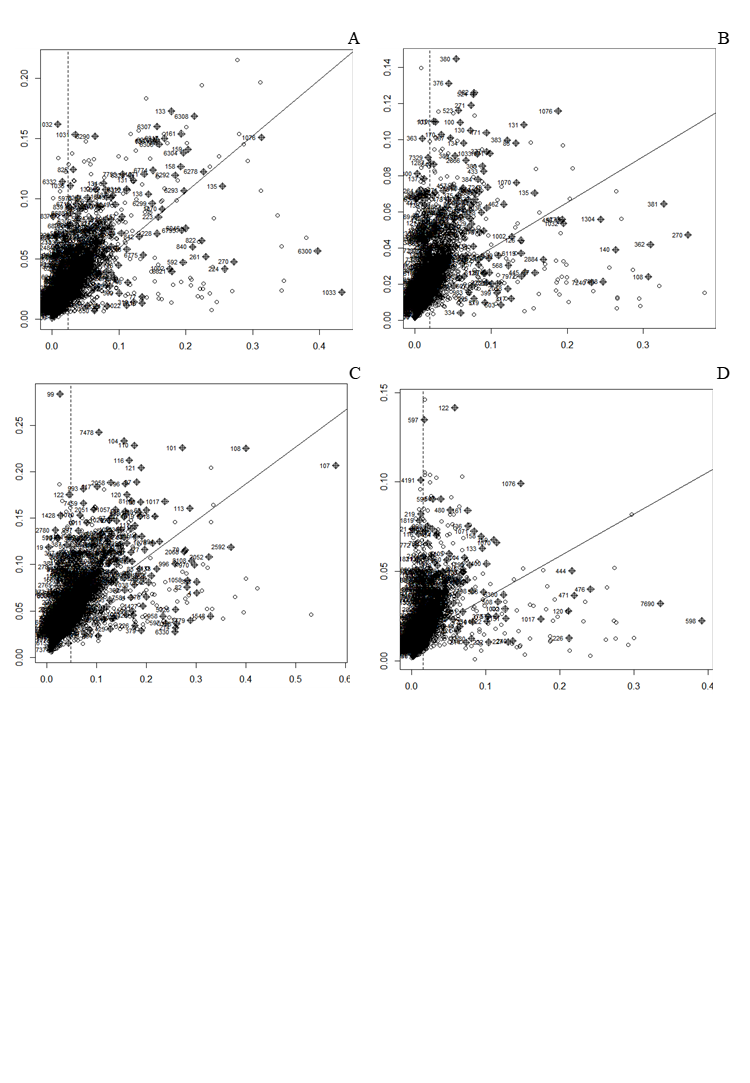

Supplement: Supplementary file 1 — Additional file 1: Supplementary Figure A1. Moran scatterplots of oral diseases in Germany. A: periodontitis, B: caries (severe), C: irreversible pulpitis, D: tooth loss. [file 12903_2024_3897_MOESM1_ESM.bmp]

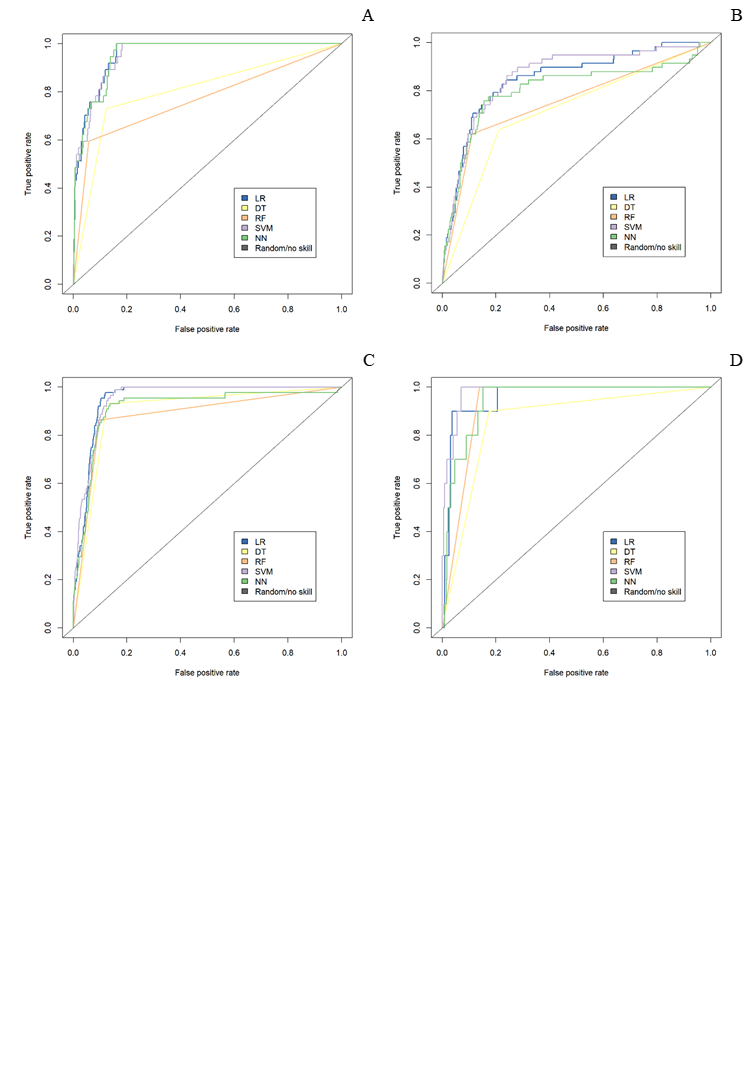

Supplement: Supplementary file 2 — Additional file 2: Supplementary Figure A2. ROC Curves for ML Classification methods. A: periodontitis, B: caries (severe), C: irreversible pulpitis, D: tooth loss. [file 12903_2024_3897_MOESM2_ESM.bmp]

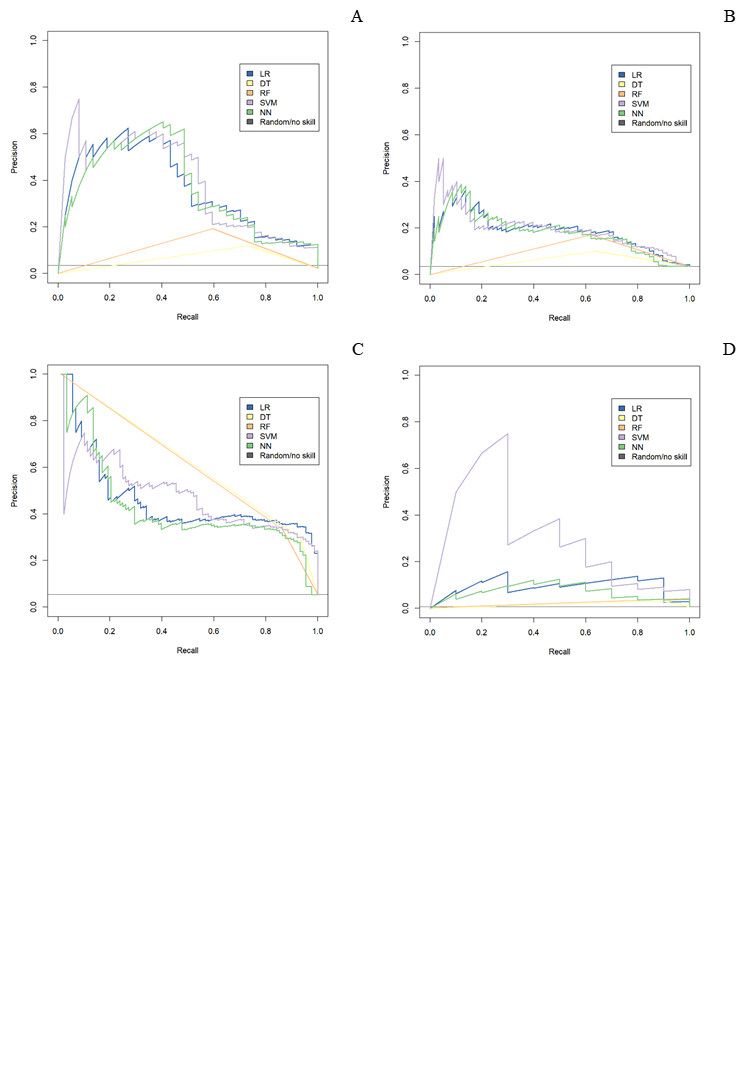

Supplement: Supplementary file 3 — Additional file 3: Supplementary Figure A3. PR AUC Curves for ML Classification methods. A: periodontitis, B: caries (severe), C: irreversible pulpitis, D: tooth loss. [file 12903_2024_3897_MOESM3_ESM.bmp]

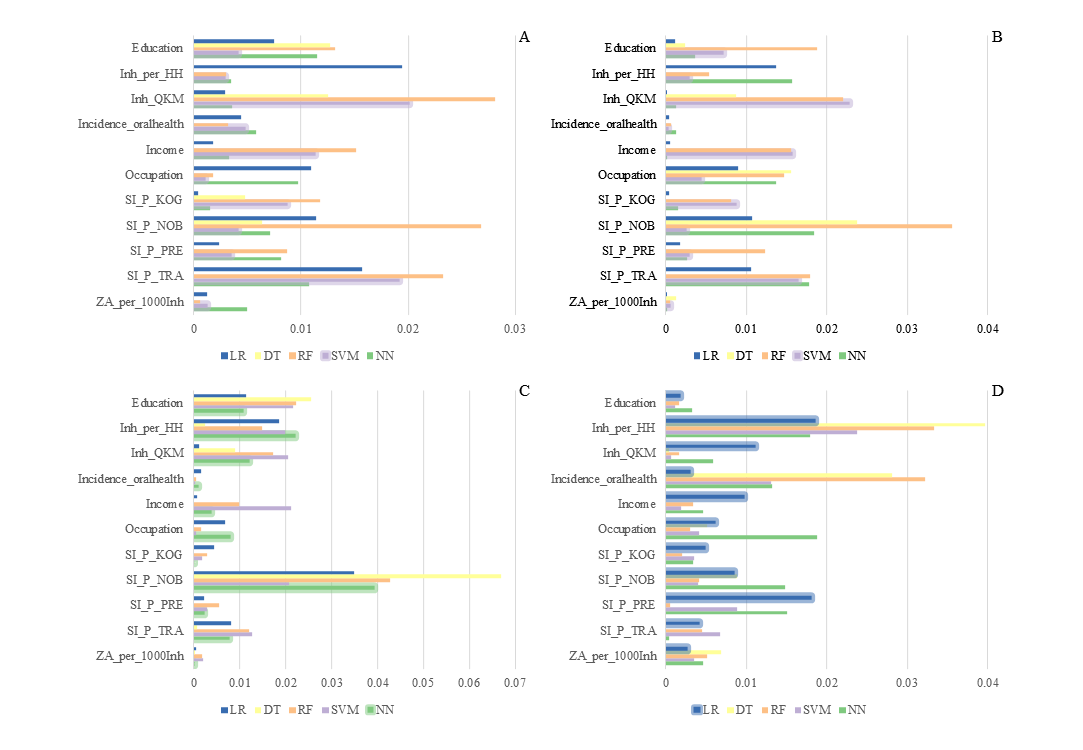

Supplement: Supplementary file 4 — Additional file 4: Supplementary Figure A4. Results of the permutation-based variable importance calculation based on root mean squared error (RMSE) loss for each disease, each model and each variable. The best performing model for each disease is highlighted. A: periodontitis, B: caries (severe), C: irreversible pulpitis, D: tooth loss. [file 12903_2024_3897_MOESM4_ESM.bmp]
