# Supplementary material for: Detecting high-risk neighborhoods and socioeconomic determinants for common oral diseases in Germany
Source: BMC Oral Health. 2024 Feb 9;24:205. doi: 10.1186/s12903-024-03897-4 (PMC11360568; doi:10.1186/s12903-024-03897-4)
Supplement: Supplementary file 5 — Additional file 5: Supplementary Table A1. Hyperparameters and Functions of Machine Learning Models for periodontitis. Supplementary Table A2. Hyperparameters and Functions of Machine Learning Models for severe caries. Supplementary Table A3. Hyperparameters and Functions of Machine Learning Models for irreversible pulpitis. Supplementary Table A4. Hyperparameters and Functions of Machine Learning Models for tooth loss. [file 12903_2024_3897_MOESM5_ESM.docx]

Supplementary Material

Detecting high-risk neighborhoods and socioeconomic determinants for common oral diseases in Germany

# Supplementary Data

Inclusion and exclusion criteria for oral diseases based on the German dental fee schedule (Gebührenordnung Zahnärzte – GOZ):

Specific GOZ for

- Periodontitis
  - Inclusion criteria:
    - patients with open therapy GOZ 4090 or 4100
    - patients with closed therapy 4070 or 4075)
    - patients with > 10 years of age
- severe caries, in which a cavity is treated and the probing depth extends down to the pulpa including direct or indirect pulp capping
  - Inclusion criteria: patients with GOZ 2330 or 2340 additionally to GOZ 2050, 2060, 2070, 2080, 2090, 2100, 2110, 2120, 2150, 2160 or 2170,
- irreversible pulpitis
  - Inclusion criteria: patients with GOZ 2360, 2390, 2400, 2410, 2420, 2430 or 2440
- tooth extraction/loss
  - Inclusion criteria: patients with GOZ 3000, 3010, 3020, 3030, 3040, 3045 or 3270
  - Exclusion criteria: all 8 teeth (wisdom teeth).

# Supplementary Figures

**Supplementary Figure A1.** Moran scatterplots of oral diseases in Germany. A: periodontitis, B: caries (severe), C: irreversible pulpitis, D: tooth loss

**Supplementary Figure A2.** ROC Curves for ML Classification methods. A: periodontitis, B: caries (severe), C: irreversible pulpitis, D: tooth loss

**Supplementary Figure A3.** PR AUC Curves for ML Classification methods. A: periodontitis, B: caries (severe), C: irreversible pulpitis, D: tooth loss

Supplementary Figure A4. Results of the permutation-based variable importance calculation based on root mean squared error (RMSE) loss for each disease, each model and each variable. The best performing model for each disease is highlighted. A: periodontitis, B: caries (severe), C: irreversible pulpitis, D: tooth loss

# Supplementary Tables

### Supplementary Table A1: Hyperparameters and Functions of Machine Learning Models for periodontitis

| **Model** | **Hyperparameters and settings** |
| --- | --- |
| Logistic Regression | - Family: Binomial - Default link function: logit. - No regularization applied |
| Decision Tree | - Method: “class” - Cp = 0.006 - Minsplit: 9 - Minbucket: 81 - Maxdepth: not limited - Xval: 10-fold CV |
| Random Forest | - Importance: TRUE - Ntree: 500 - Mtry: 3, - Nodesize: 1 for classification |
| Support Vector Machines | - Kernel: Radial - Cost: 1 - Gamma: 0.09 |
| Neural Network | - size=5 - decay=0.001 - maxit=150 |

### Supplementary Table A2: Hyperparameters and Functions of Machine Learning Models for severe caries

| **Model** | **Hyperparameters and settings** |
| --- | --- |
| Logistic Regression | - Family: Binomial - Default link function: logit. - No regularization applied |
| Decision Tree | - Method: “class” - Cp = 0.0045 - Minsplit: 11 - Minbucket: 81 - Maxdepth: not limited - Xval: 10-fold CV |
| Random Forest | - Importance: TRUE - Ntree: 500 - Mtry: 3, - Nodesize: 1 for classification |
| Support Vector Machines | - Kernel: Radial - Cost: 1 - Gamma: 0.09 |
| Neural Network | - size=6 - decay=0 - maxit=100 |

### Supplementary Table A3: Hyperparameters and Functions of Machine Learning Models for irreversible pulpitis

| **Model** | **Hyperparameters and settings** |
| --- | --- |
| Logistic Regression | - Family: Binomial - Default link function: logit. - No regularization applied |
| Decision Tree | - Method: “class” - Cp = 0.004 - Minsplit: 12 - Minbucket: 81 - Maxdepth: not limited - Xval: 10-fold CV |
| Random Forest | - Importance: TRUE - Ntree: 500 - Mtry: 3, - Nodesize: 1 for classification |
| Support Vector Machines | - Kernel: Radial - Cost: 0.8 - Gamma: 0.09 |
| Neural Network | - size=5 - decay=0 - maxit=100 |

### Supplementary Table A4: Hyperparameters and Functions of Machine Learning Models for tooth loss

| **Model** | **Hyperparameters and settings** |
| --- | --- |
| Logistic Regression | - Family: Binomial - Default link function: logit. - No regularization applied |
| Decision Tree | - Method: “class” - Cp = 0.005 - Minsplit: 10 - Minbucket: 81 - Maxdepth: not limited - Xval: 10-fold CV |
| Random Forest | - Importance: TRUE - Ntree: 500 - Mtry: 3, - Nodesize: 1 for classification |
| Support Vector Machines | - Kernel: Radial - Cost: 0.8 - Gamma: 0.09 |
| Neural Network | - size=5 - decay=0.001 - maxit=100 |
